# Editorial: Autophagy and Ageing: Ideas, Methods, Molecules

**DOI:** 10.3389/fcell.2020.00141

**Published:** 2020-02-28

**Authors:** Tassula Proikas-Cezanne, Nicholas T. Ktistakis

**Affiliations:** ^1^Interfaculty Institute of Cell Biology (IFIZ), Eberhard Karls University Tübingen, Tübingen, Germany; ^2^Babraham Institute, Signaling Programme, Babraham, United Kingdom

**Keywords:** autophagy, ageing, mitophagy, lifespan, health, model organisms

Ever since it was first discussed in evolutionary terms by Haldane (1941), Medawar (1952), and Williams (1957), [reviewed in Partridge and Gems (2006)] aging has become a focus of much current research interest, especially following discoveries pointing to molecular, genetic, and biochemical hallmarks that control its progression and severity (López-Otín et al., 2013). Amongst the many cellular processes that provide physiological connections to the aging process, autophagy is perhaps one of the most compelling (Rubinsztein et al., 2011; Nakamura and Yoshimori, 2018). There have been over 1,100 reviews since the discovery of autophagy related (ATG) genes by Tsukada and Ohsumi (1993)−300 or so in the last year alone—linking the process of aging to some aspect of autophagy! Even within the ever-expanding autophagy opus, such numbers are impressive, and perhaps unique. Articles published in this Frontiers collection that we edited exemplify this trend.

The pathway of autophagy allows organisms to survive periods of starvation of moderate duration, and to eliminate from their cell cytosol unwanted material not easily handled by the proteasome (Morishita and Mizushima, 2019). Both of these autophagy functions, nutrient replenishment and quality control, are positive contributors to healthy physiology, although they can also promote survival of cancer cells during uncontrolled growth. As part of a process contributing to healthy physiology, autophagy has been linked to healthy aging in correlative as well as causally linked ways (reviewed in Hansen et al., 2018). In terms of the former, various organisms show a decline of autophagy with aging; in terms of the latter, the majority of genetic manipulations that extend lifespan in model organisms require an intact autophagy pathway. Such concepts are extensively discussed by Stead et al., with special emphasis on ways that modulation of autophagy can be beneficial for healthy aging. A specific focus on lessons learned from Drosophila on the functional connection between autophagy and aging is provided by Marusz et al. However, what is less clear from the data available so far is whether enhanced autophagy *on its own* is necessary and sufficient to extend life span. Some studies strongly support the idea that this is the case (reviewed in Hansen et al., 2018), but in our opinion many more studies underpinned by robust autophagy measurements and more mechanistic molecular detail are required to make this point clearly.

How does autophagy engage with the aging process? At one level this is related to caloric restriction, one of the most reliable ways to extend life span in model organisms (López-Otín et al., 2013). Autophagy is strongly activated during caloric restriction in order to provide nutrients from recycling, and it becomes an essential organismal response during periods of extreme starvation such as, e.g., the early neonatal period (Kuma et al., 2004). Several articles in this collection discuss the relation between autophagy and nutrient availability. Plummer and Johnson examine how restriction of methionine, one of the most effective dietary restrictions that extend lifespan, stimulates autophagy, and especially the autophagic elimination of mitochondria, in order to significantly extend yeast chronological lifespan. Bareja et al. discuss the signaling pathways affecting aging and how they can be beneficially manipulated starting from something as simple as increased exercise. A master regulator of nutrient sensing is mTOR, and Schmeisser and Parker discusses how interfering with this particular signaling pathway can have very complicated effects. At another level, autophagy is a major pathway counteracting the accumulation of damaged (and damaging) material in cells as a function of age. Such material can be dysfunctional mitochondria, accumulated lipids and, in the case of neurons, aggregated proteins potentially causing neurodegeneration (Gatica et al., 2018). This type of selective autophagy of lipids in the context of aging and various pathological conditions is discussed in this collection by Kounakis et al. Finally, it is important to remember that aging is a very complex process that affects with different severity and timing the various bodily tissues. Along those lines, the relation between autophagy and aging of specific cells and tissues is also addressed in this collection. Macian reviews the role of autophagy in T cell function and immunosenescence, a very interesting phenomenon in immune cells, whereas Eckhart et al. discuss how the various cells of the skin activate autophagy and the connection of this process to skin aging.

The developmental theory of aging proposes that the optimization of developmental programs necessary for reproductive fitness has the unwanted consequence of also causing aging when these programs continue to operate past reproductive age (de Magalhães and Church, 2005). In this view aging is not under any selective pressure, and it was a wide-held view some time ago that the genetic analysis of this process may be impossible (Johnson, 2002). The subsequent isolation of long-lived mutants, mapping to single genes, dispelled the skepticism (Kenyon, 2010). All the while, the developmental theory of aging continues to offer a compelling framework to think about the mechanisms that drive this process (see also the discussion by Schmeisser and Parker. Of note, several genes whose downregulation enhances lifespan are also shown to be important for early development (Kenyon, 2010). Autophagy connects in an interesting way to the developmental theory of aging. It is a survival mechanism that becomes essential in periods of neonatal starvation, and in this function it makes sense to be evolutionarily conserved. At the same time, the autophagic process is also beneficial, as we have discussed here, as a countermeasure for aging, but this function cannot be selected for in evolution since it benefits organisms past their reproductive stage. The interesting point is that autophagy may be an example of a process whose benefit in later life is inadvertently maintained by evolution based on its importance during the reproductive stage. In this way, manipulations that enhance autophagy for life span extension may be well-tolerated with minimal side effects.

We hope that the articles collected in this volume will stimulate further hypothesis-driven experimental work detailing the role of autophagy in the aging process.

## Author Contributions

TP-C and NK wrote and edited the manuscript.

### Conflict of Interest

The authors declare that the research was conducted in the absence of any commercial or financial relationships that could be construed as a potential conflict of interest.
